# Retinoic Acid Alleviates Cisplatin-Induced Acute Kidney Injury Through Activation of Autophagy

**DOI:** 10.3389/fphar.2020.00987

**Published:** 2020-07-03

**Authors:** Junxia Wu, Canbin Zheng, Xin Wan, Mingjun Shi, Kathryn McMillan, Jenny Maique, Changchun Cao

**Affiliations:** ^1^ Department of Nephrology, Sir Run Run Hospital, Nanjing Medical University, Nanjing, China; ^2^ Department of Nephrology, Nanjing First Hospital, Nanjing Medical University, Nanjing, China; ^3^ Department of Internal Medicine, University of Texas Southwestern Medical Center, Dallas, TX, United States; ^4^ Department of Orthopedic and Microsurgery, The First Affiliated Hospital of Sun Yat-sen University, Guangzhou, China; ^5^ Department of Molecular Biology, University of Texas Southwestern Medical Center, Dallas, TX, United States

**Keywords:** retinoic acid, cisplatin, acute kidney injury, autophagy, apoptosis

## Abstract

Cisplatin-induced acute kidney injury (CIAKI) is a common complication in patients receiving cisplatin-based chemotherapy. But the effective therapies for CIAKI are not available. Retinoic acid (RA), the main derivative of vitamin A, has the potential to reduce inflammation and fibrosis in renal injury. However, the effect and mechanism of RA on CIAKI are still unclear. The aim of this study is to investigate whether RA can alleviate CIAKI through activation of autophagy. In this study, we evaluated the effect of RA, RA’s effect on autophagy and apoptosis after cisplatin-induced injury on renal tubular epithelial cells (RTECs) by LDH assay, immunoblotting and TUNEL staining. Then we established Atg5^flox/flox^:Cagg-Cre mice in which Cagg-Cre is tamoxifen inducible, and Atg5 is conditional deleted after tamoxifen injection. The effect of RA and RA’s effect on autophagy on CIAKI model were evaluated by biochemical assessment, hematoxylin and eosin (HE) staining, and immunoblotting in the control and autophagy deficient mice. *In vitro*, RA protected RTECs against cisplatin-induced injury, activated autophagy, and inhibited cisplatin-induced apoptosis. *In vivo*, RA attenuated cisplatin-induced tubular damage, shown by improved renal function, decreased renal cast formation, decreased NGAL expression, and activated autophagy in the control mice. Furthermore, the nephrotoxicity of cisplatin was aggravated, and the protective effect of RA was attenuated in autophagy deficient mice, indicating that RA works in an autophagy-dependent manner on CIAKI. RA activates autophagy and alleviates CIAKI *in vivo* and *in vitro*.Thus RA may be a renoprotective adjuvant for cisplatin-based chemotherapy.

## Introduction

Acute kidney injury (AKI) is a syndrome characterized by a rapid loss of the kidney’s excretory function and a defect in the excretion of water, salts, and metabolic products including creatinine (Bellomo et al., 2012; Barasch et al., 2017). The occurrence of AKI is more than 36% on the day after admission to an intensive care unit (Bagshaw et al., 2008), and the incidence is greater than 60% during intensive care unit admission (Hoste and Kellum, 2006). Patients with AKI show high morbidity and mortality (Waikar et al., 2008; Thomas et al., 2015; Yang et al., 2015). Cisplatin-incuced AKI (CIAKI) is a common complication of administering cisplatin, a chemotherapeutic agent used to treat solid tumors (Wang and Lippard, 2005). However, effective therapies for CIAKI and detailed mechanisms still need further exploration.

Retinoic acid (RA), the main derivative of vitamin A, plays an important role in tissue development and differentiation. RA has cytoprotective effects in the kidney (Evans and Kaye, 1999; Kishimoto et al., 2011), and RA agonists reduce injury and inflammation in ischemia–reperfusion AKI and unilateral ureteral obstruction (UUO) models (Wagner et al., 2000; Lehrke et al., 2002; Perez et al., 2004; Kishimoto et al., 2011; Ratnam et al., 2011; Balasubramanian et al., 2012; Chiba et al., 2016). However, RA’s effect on CIAKI needs to be elucidated. We examined the effect of RA on cisplatin-induced renal tubular epithelial cells (RTECs) injury and found that RA protected against cisplatin-induced RTECs injury *in vitro*. On the basis of *in vitro* results, the effect of RA using a CIAKI murine model was further evaluated.

Autophagy is a highly conserved multistep pathway for maintaining intracellular homeostasis by degrading and recycling damaged macromolecules and organelles (Kaushal and Shah, 2016). Previous research has demonstrated that autophagy guarded against CIAKI (Takahashi et al., 2012). *In vitro*, we also found that activating autophagy protected against cisplatin-induced RTECs injury, while inhibiting autophagy aggravated RTECs injury and that RA activated autophagy. Thus, we hypothesized that RA alleviated CIAKI through activation of autophagy. Atg 5 is one of the critical genes involved in the autophagy process (Kaushal and Shah, 2016; Fernandez et al., 2018). Based on these findings, we established Atg5^flox/flox^:Cagg-Cre mice in which conditional deletion of Atg5 was obtained after tamoxifen treatment to explore whether RA alleviated CIAKI through activation of autophagy. Then the effect of RA and RA’s effect on autophagy on CIAKI model in the control and autophagy deficient mice were evaluated. As expected, we demonstrated that RA confers renoprotective effects on CIAKI *via* an autophagy dependent mechanism.

## Results

### RA Alleviated CIAKI in Atg5^flox/flox^ or Cagg-Cre Mice

**Figure 1:**
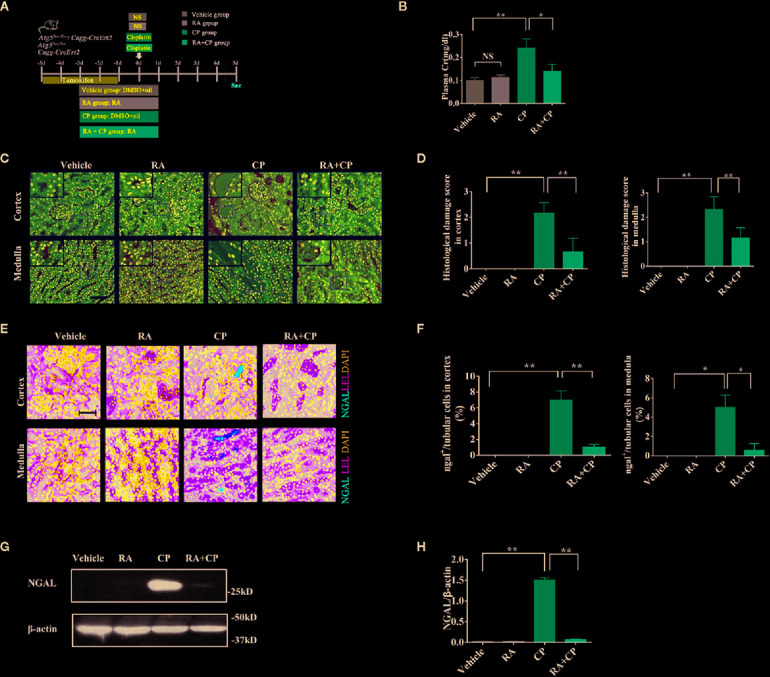
RA alleviated CIAKI in Atg5^flox/flox^ or Cagg-Cre mice. **(A)** Male and female Atg5^flox/flox^, CaggCre, and Atg5^flox/flox^:CaggCre mice were divided into four groups randomly (n = 6). Mice were injected with cisplatin with or without RA in different groups as the flowchart showed. **(B)** Plasma creatinine was measured. **(C)** Kidney morphology of the cortex and medulla (HE, magnification ×400) showed tubular necrosis (depicted by red arrows) and cast formation (depicted by red asterisks). The RA pretreatment group showed significantly less cast formation than the cisplatin group. Scale bar, 100 μm. **(D)** Pathological scores of tubular damage in the cortex and medulla of each group. **(E)** Representative immunofluorescence images of NGAL magnification ×600. NGAL (red), LEL (green), DAPI (blue). Scale bar, 50 μm. **(F)** Quantification of immunofluorescence images of NGAL. **(G)** Whole-tissue lysates of kidney were collected for immunoblot analysis of NGAL and *β*-actin. **(H)** Densitometry of NGAL signals. Data in B, D, F, and H are expressed as the means ± SDs, *P < 0.05, **P < 0.01, NS, not significant.

Male and female Atg5^flox/flox^ or Cagg-Cre and Atg5^flox/flox^:Cagg-Cre mice were grouped randomly as shown in the flowchart (n = 6) (**Figure 1A**). Plasma creatinine was measured as a marker of renal function. We established the CIAKI model by retro-orbital injection of 15 mg/kg cisplatin. In Atg5^flox/flox^ or Cagg-Cre mice, the plasma creatinine level of the cisplatin group increased significantly compared with those of the vehicle group; however, compared with those of the cisplatin group, the plasma creatinine level of the RA treatment group decreased significantly (**Figure 1B**, P < 0.05). In Atg5^flox/flox^ or Cagg-Cre mice, the pathological scores of tubular damage in the cortex and medulla of the RA treatment group decreased significantly compared with those of the cisplatin group (**Figures 1C, D**, P < 0.01). Compared with that in the cisplatin group, the expression level of NGAL in the RA treatment group decreased significantly, as shown by immunoblotting and immunofluorescence analysis in Atg5^flox/flox^ or Cagg-Cre mice (**Figures 1E–H**, P < 0.05). These results demonstrated that RA alleviates CIAKI in Atg5^flox/flox^ or Cagg-Cre mice.

### RA Activated Autophagy in the Kidney in Atg5^flox/flox^ or Cagg-Cre Mice

**Figure 2:**
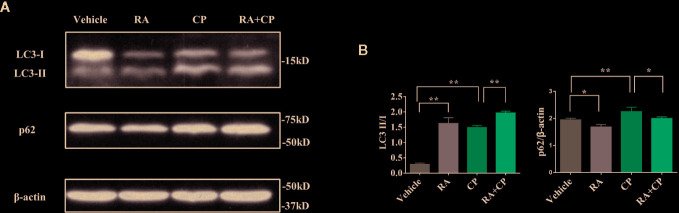
RA activated autophagy in the kidney in Atg5^flox/flox^ or Cagg-Cre mice. **(A)** Whole-tissue lysates of kidney were collected for immunoblot analysis of p62, LC3-II, LC3-I, and *β*-actin. **(B)** Quantitative data of the p62 level and LC3-II/LC3-I ratio. Data in B are expressed as the means ± SDs, *P < 0.05, **P < 0.01.

In Atg5^flox/flox^ or Cagg-Cre mice, compared with the vehicle group, the RA group showed an increased LC3-II/I ratio and a decreased p62 level by immunoblotting. Compared with the cisplatin group, the RA treatment group also showed an increased LC3-II/I ratio and a decreased p62 level by immunoblotting (**Figures 2A, B**, P < 0.05). This finding suggests that RA activates autophagy in the kidney in Atg5 flox/flox or Cagg-Cre mice.

### CIAKI Was Aggravated and the Protective Effect of RA Was Attenuated In Atg5^flox/flox^:Cagg-Cre Mice

**Figure 3:**
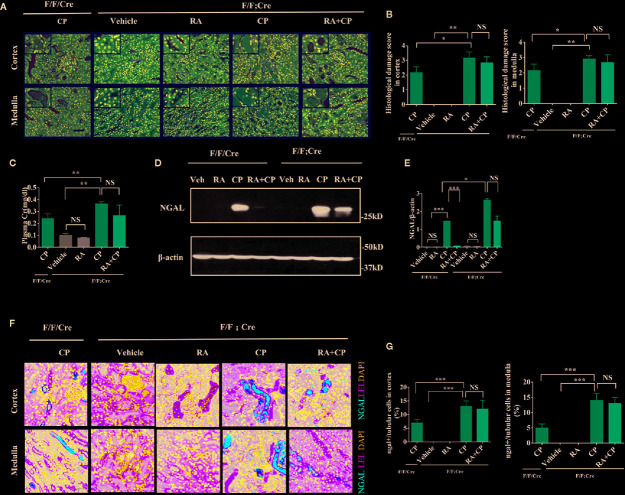
CIAKI was aggravated and the protective effect of RA was attenuated in Atg5 ^flox/flox^:Cagg-Cre mice.**(A)** Kidney morphology of the cortex and medulla (HE staining, magnification ×400) showed tubular necrosis (depicted by red arrows) and cast formation (depicted by red asterisks). Scale bar, 100 μm. **(B)** Pathological scores of tubular damage in the cortex and medulla of each group. **(C)** Blood samples were collected for measurement of plasma creatinine level. Data are expressed as the means ± SDs (n = 6). **(D)** Whole-tissue lysates of kidney were collected for immunoblot analysis of NGAL and *β*-actin. **(E)** Densitometry of NGAL signals. **(F)** Representative immunofluorescence images of NGAL magnification ×600. NGAL (red), LEL (green), DAPI (blue). Scale bar, 50 μm. **(G)** Quantification of immunofluorescence images of NGAL of each group. Data in B, C, E, and G are expressed as the means ± SDs, *P < 0.05, **P < 0.01, ***P < 0.001, NS, not significant.

After cisplatin injection, Atg5^flox/flox^:Cagg-Cre mice had more severe tissue damage in the kidney, shown by higher damage scores in the renal cortex and medulla than Atg5^flox/flox^ or Cagg-Cre mice(**Figures 3A, B**, P < 0.05), while tissue damage did not alleviate significantly in the RA treatment group compared with that in the cisplatin group of Atg5^flox/flox^:Cagg-Cre mice (**Figures 3A, B**, P > 0.05). Atg5 ^flox/flox^:Cagg-Cre mice had more severe loss of renal function, which was indicated by higher plasma creatinine than Atg5^flox/flox^ or Cagg-Cre mice in the cisplatin group (**Figure 3C**, P < 0.01). However, the plasma creatinine level of the RA treatment group showed no significant change compared with that of the cisplatin group in Atg5^flox/flox^:Cagg-Cre (**Figure 3C**, P > 0.05). Compared with that of Atg5^flox/flox^ or Cagg-Cre mice, the expression level of NGAL in the cisplatin group of Atg5^flox/flox^:Cagg-Cre mice increased significantly (**Figures 3D–G**, P < 0.05) and that in the RA treatment group of Atg5^flox/flox^:Cagg-Cre mice did not change significantly, as shown by immunoblotting and immunofluorescence analysis (**Figures 3D–G**, P > 0.05).

### RA or Autophagy Protected Against Cisplatin-Induced RTECs Injury *In Vitro*

**Figure 4:**
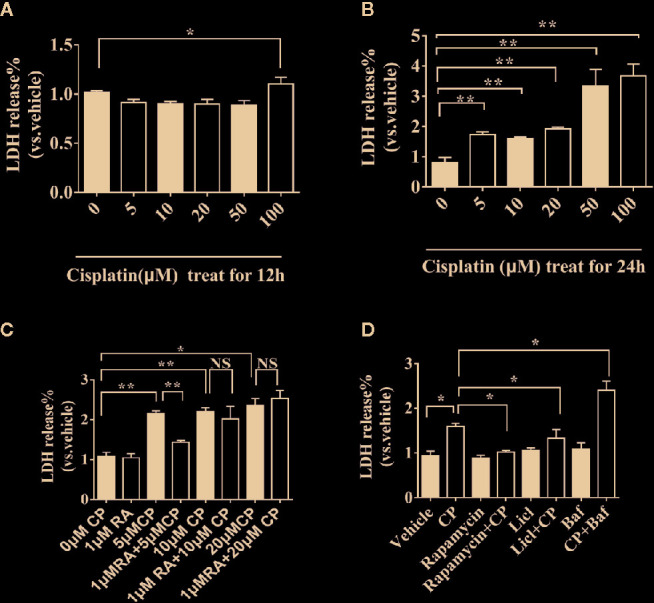
RA or autophagy protected against cisplatin-induced RTECs injury *in vitro*. **(A)** NRK cells were treated with different doses (0–100 μM) of cisplatin for 12 h. **(B)** NRK cells were treated with different doses (0–100 μM) of cisplatin for 24h. **(C)** NRK cells were treated with 0-20 μM of cisplatin for 24h with or without 1 μM RA. **(D)** NRK cells were treated with 5 μM cisplatin with autophagy inducer (0.5 μM rapamycin) or autophagy inhibitor (200 nM Bafilomycin A1). Data are expressed as the means ± SDs *P < 0.05, **P < 0.01, NS, not significant.

NRK cells were treated with 0, 5, 10, 20, 50, or 100 μM cisplatin for 12 h or 24 h (**Figures 4A, B** **)**. Only 0–100 μM cisplatin treatment for 24 h showed dose-dependent toxicity (P < 0.01), and 50 μM cisplatin caused cell death. Thus, 24 h was chosen as the suitable treatment time, and 0–20 μM cisplatin was chosen for further research. We found that RA could protect against cisplatin-induced cell injury at 5 μM, as shown by the decreasing LDH value compared with that of the vehicle group (**Figure 4C**, P < 0.01). Therefore, we chose 5 μM cisplatin for further experiments. Cells were treated with 5 μM cisplatin and autophagy inducer (10 mM LiCl, 0.5 μM rapamycin) or inhibitor (200 nM Bafilomycin A1). LiCl and rapamycin decreased cisplatin-induced LDH release while Bafilomycin A1 increased cisplatin-induced LDH release (**Figure 4D**, P < 0.05). The results show that upregulating autophagy can alleviate cisplatin-induced cell injury, while downregulating autophagy aggravates cisplatin-induced cell injury.

### RA Induced Autophagy After Cisplatin-Induced RTECs Injury ***In Vitro***

**Figure 5:**
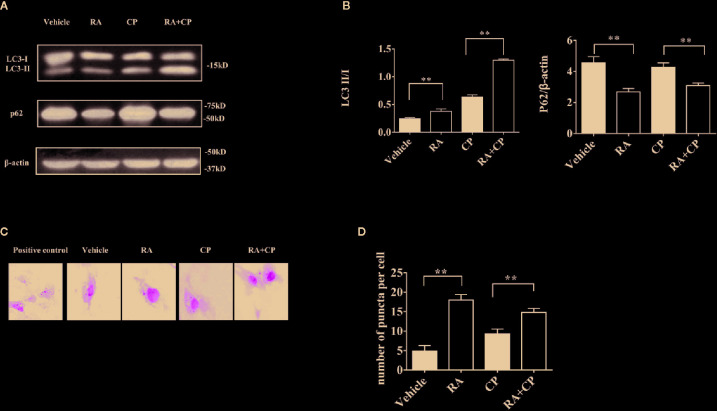
RA induced autophagy after cisplatin-induced RTECs injury *in vitro*. **(A)** NRK cells were treated with 5 μM cisplatin and/or 1 μM RA or Bafilomycin A1. Whole-cell lysates of NRK were collected for immunoblot analysis of p62, LC3-II, LC3-I and β-actin. **(B)** Densitometry of p62 and LC3-II/LC3-I ratio of three independent experiments. **(C)** OK cells were seeded on glass coverslips and transfected with GFP-LC3 plasmid. OK cells were treated for 24 h with 5 μM cisplatin with or without 1 µM RA after transfection for 24 h. Scale bar, 10 μm. **(D)** Quantification of the number of GFP-LC3 puncta (Six cells analyzed per sample). Data in B and D are expressed as the means ± SDs, **P < 0.01.

NRK cells were treated with 0 or 5 μM cisplatin with or without 1 μM RA for 24 h. Compared with the vehicle group, the RA group showed increased LC3-I to LC3-II conversion and a decreased p62 level, while compared with the cisplatin group, the RA treatment group also showed increased LC3-I to LC3-II conversion and a decreased p62 level (**Figures 5A, B**, P < 0.01). Compared with the vehicle group, the RA group showed an increased number of autophagy puncta. Furthermore, compared with the cisplatin group, the RA treatment group showed an increased number of autophagy puncta (**Figures 5C, D**, P < 0.01). These results indicate that RA activates autophagy after cisplatin-induced RTECs injury.

### RA Inhibited Apoptosis After Cisplatin-Induced RTECs Injury ***In Vitro***

**Figure 6:**
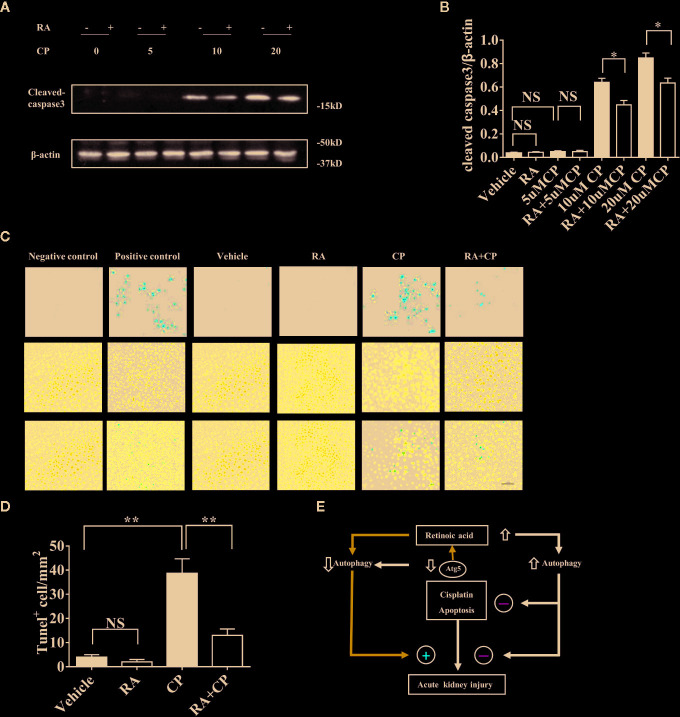
RA inhibited apoptosis after cisplatin-induced RTEC injury *in vitro*. **(A)** NRK cells were treated with 0–20 μM cisplatin with or without 1 µM RA for 24 h. Whole-cell lysates of NRK cells were collected for immunoblot analysis of cleaved-caspase-3 and *β*-actin. **(B)** Densitometry of cleaved-caspase-3 signals. **(C)** Cells were fixed with 4% PFA for 10 min and were treated according to the TUNEL kit instructions. Red cells were TUNEL positive cells and the nuclei were stained with DAPI (blue). **(D)** Quantification of the number of TUNEL-positive cells for every mm^2^ under the microscope. **(E)** Proposed working model of the effect of retinoic acid on autophagy in cisplatin induced AKI. Retinoic acid increases autophagic flux and protects kidney from cisplatin induced injury and exerts antiapoptosis effect. When Atg5 is deficient, the basal autophagy level is downregulated and cisplatin induced AKI is aggravated and the protective effect of retinoic acid is attenuated. Thus retinoic acid protects the kidney against cisplatin-induced injury which is dependent on the regulation of autophagy. Data in B and D are expressed as the means ± SDs, *P < 0.05 **P < 0.01 NS, not significant.

After 10 or 20 μM cisplatin treatment, the expression level of cleaved-caspase-3 increased significantly compared with that in the vehicle treatment, and RA decreased the cleaved-caspase 3 level induced by cisplatin treatment as shown by immunoblotting (**Figures 6A, B**, P < 0.05). For the cisplatin group, a 10 μM concentration was chosen for the TUNEL experiment, and RA treatment decreased the apoptosis ratio induced by cisplatin (**Figures 6C, D**, P < 0.01). These findings show that RA inhibited apoptosis after cisplatin-induced RTECs injury. And the proposed working model of the effect of retinoic acid on autophagy in cisplatin induced AKI was showed in **Figure 6E**.

## Discussion

The mechanisms underlying CIAKI remain largely unclear. Pathological features of CIAKI include proximal tubule injury, inflammation, oxidative stress, and vascular injury in the kidney. In addition, proximal tubule injury involves several mechanisms, such as autophagy and apoptosis (Wei et al., 2007; Pabla and Dong, 2008). RA is an active metabolite form of vitamin A (Kedishvili, 2016). Studies have demonstrated that RA has a renoprotective effect on kidney disease, especially glomerulonephritis (Mallipattu and He, 2015; Dai et al., 2017). Previous research has demonstrated that autophagy is a target for RA, and RA promotes autophagosome maturation(Rajawat et al., 2010). We thus assumed that RA could protect against CIAKI *via* activation of autophagy.

During autophagy, there are several distinct stages: vesicle nucleation, vesicle elongation and completion, fusion of the autophagosome with the lysosome to form an autolysosome, lysates of the autophagosome, and breaking down of its contents (Moscat and Diaz-Meco, 2011). Atg5 conjugates to Atg12 to form the Atg5–Atg12 complex and is involved in the elongation of membranes that evolve into autophagosomes during the autophagy process (Mizushima et al., 2003; Yang and Klionsky, 2010; Mizushima and Komatsu, 2011; Rubinsztein et al., 2012; Feng et al., 2014); however, mice deficient in Atg5 will die within 1 day after birth (Yoshii et al., 2017). Therefore, we established a conditional Atg5 knockout mouse model by intraperitoneal injection of tamoxifen to induced heterozygote deletion effect *via* the Cre-loxP system as described previously (Boneva et al., 2016) to investigate the role of RA and autophagy on CIAKI in Atg5^flox/flox^, Cagg-Cre and Atg5^flox/flox^:Cagg-Cre mice. We demonstrated the renoprotective effect of RA on CIAKI in Atg5^flox/flox^ and Cagg-Cre mice, shown by improved renal function, less kidney tissue damage by HE staining, and a decrease in NGAL level by immunoblotting and immunofluorescence, while in Atg5^flox/flox^:Cagg-Cre mice, CIAKI was aggravated, shown by deteriorating renal function, more kidney tissue damage and a higher level of NGAL. And the protective effect of RA on CIAKI was attenuated.

In our research, RA activates autophagy, which was indicated by an increased LC3-II/I ratio and a decreased level of p62 by immunoblotting in Atg5^flox/flox^ or Cagg-Cre mice and in kidney cells. We were not able to obtain transgenic GFP-LC3 mice to observe autophagy punta *in vivo*; therefore, we transiently transfected the GFP-LC3 plasmid into kidney cells and observed autophagy punta as indicated by more LC3-positive puncta after RA treatment. This study demonstrated that RA activated autophagy and inhibited apoptosis after cisplatin-induced injury, as shown by reduced cleaved-caspase-3 by immunoblotting and less TUNEL-positive renal epithelial cells. These findings suggest that RA stimulates autophagy and blocks the induction of apoptosis-associated caspase activation. However, the detailed mechanisms by which RA regulates autophagy and apoptosis remain to be further explored.

Above all, our study identified the renal protective effect of a low dose of RA on CIAKI, and RA activated autophagy *in vivo* and *in vitro*. We also demonstrated that CIAKI was aggravated while RA’s protective effect was attenuated when autophagy was inhibited *in vivo* and *in vitro*. Thus, RA alleviates CIAKI by activating autophagy. While on cancer cells, RA has differentiation-inducing, antiproliferative, and proapoptotic effects, which are different from the effects on normal cells (di Masi et al., 2015). Previous studies have demonstrated that RA in combination with cisplatin showed a synergistic effect in decreasing cell viability in CD44(+) cancer stem cells (Najafzadeh et al., 2015). However, our study lacks *in vivo* and *in vitro* experiments in tumor-bearing mice and cancer cells. In the future, further studies will be needed before RA can be evaluated as a renoprotective adjuvant for cisplatin-based chemotherapy.

## Materials and Methods

### Ethics Statement

We strictly followed the Guide for the Care and Use of Laboratory Animals by the National Institutes of Health, and all protocols for all the mice in the experiment were approved by the Institutional Animal Care and Use Committee at the University of Texas Southwestern Medical Center. All the mice were anesthetized and euthanized with isoflurane, and every effort was made to minimize the animal suffering.

### Murine Model of CIAKI

All mice were from a 129 SI/SVlmj (129SV) background at twelve-week-old for each group. Atg5^flox/flox^ mice were crossed with Cagg-CreErt2 transgenic mice expressing Cre recombinase under the control of a Cagg promoter to generate Atg5^flox/flox^:Cagg-Cre mice. Atg5^flox/flox^ mice and Cagg-CreErt2 transgenic mice were all provided by the O’Brien Kidney Research Center at the University of Texas Southwestern Medical Center, USA. And characterization of the Atg5^flox/flox^:Cagg-Cre mice model was described in **Figure S1**. Mice were housed in the animal room, which was temperature- and humidity-controlled with a 12:12 h light–dark cycle and were given free access to tap water and standard rodent chow before and during the experiment.

Mice were used for the experiment after their genotypes were confirmed, and they were divided into four groups randomly: Vehicle group, RA group, Cisplatin (CP) group, and RA + CP group. (1) The vehicle group mice were retro-orbitally injected once with the same volume of normal saline that the cisplatin group received after injection with tamoxifen. As RA was dissolved with DMSO and diluted in peanut oil, mice were also intraperitoneally injected with the same volume of DMSO and oil as the RA group on the third day of tamoxifen injections. (2) The RA group mice were intraperitoneally injected with RA on the third day of tamoxifen injections. They were also retro-orbitally injected once with the same volume of normal saline as the cisplatin group received after injection with tamoxifen. (3) The CP group mice were retro-orbitally injected once with cisplatin after injection with tamoxifen and were injected with the same volume of DMSO and oil as the RA group on the third day of tamoxifen injections. (4) The RA + CP group mice were intraperitoneally injected with RA on the third day of tamoxifen injections. They were also retro-orbitally injected once with cisplatin after injection with tamoxifen. Tamoxifen was intraperitoneally injected into mice at 50 mg/kg/d, and RA was intraperitoneally injected into mice at 2.5 mg/kg/d for 5 consecutive days. All the mice were sacrificed 5 days after cisplatin or normal saline injection. Under isoflurane anesthesia, blood samples were collected for measurement of plasma creatinine level. Each kidney was harvested and cut into three pieces. One piece was saved in 4% formaldehyde for paraffin sectioning, and the remaining pieces were instantly frozen in liquid nitrogen and stored in a −80°C freezer for further molecular analysis. Paraffin sections were used for hematoxylin and eosin (HE) staining and immunofluorescence staining for neutrophil gelatinase-associated lipocalin (NGAL). Pathological scoring of tubular damage was evaluated according to the percentage of tubules that showed tubular necrosis and cast formation as follows: 0 = no damage; 1 = mild (less than 10%); 2 = moderate (10 to 25%); 3 = severe (25 to 50%); 4 = very severe (50 to 75%); 5 = extensive damage (more than 75%). Six randomly chosen fields (×200) were evaluated for each specimen, and an average score was calculated. Histopathological changes were blindly scored by a pathologist.

### Reagents and Materials

The following primary antibodies were used for immunoblotting or immunofluorescence: rabbit monoclonal anti-Atg5 and mouse anti-p62 (Novus Biologicals, Littleton, CO, USA), mouse monoclonal anti-*β*-actin (MilliporeSigma, St. Louis, MO, USA), goat monoclonal anti-NGAL (R&D Systems, Minneapolis, MN, USA), rabbit anti-LC3B and anti-Cleaved Caspase-3 (Cell Signaling Technology, Danvers, MA, USA). Secondary antibodies were purchased from Invitrogen (Carlsbad, CA, USA). RA, Triton X-100, tamoxifen, peanut oil, Baf, LiCl, bovine serum albumin (BSA), and cisplatin were purchased from Sigma (St. Louis, MO, USA). Rapamycin was obtained from LC Laboratories (Woburn, MA, USA). Cisplatin was dissolved in normal saline to a concentration of 2 mg/ml, and tamoxifen was dissolved in peanut oil to a concentration of 10 mg/ml. RA was dissolved in DMSO and diluted with peanut oil to 0.375 mg/ml. Baf, rapamycin and LiCl were dissolved in DMSO, and the working concentrations were 200 nM, 0.5 μM, and 10 mM, respectively, for cell culture (Shi et al., 2016). The final volume of DMSO in cell culture media never exceeded 1/100. Donkey anti-goat IgG and Alexa Fluor 568 were bought from Invitrogen, and Lycopersicon esculentum lectin (LEL) and DAPI were purchased from Vector Laboratories (Burlingame, CA, USA) and MilliporeSigma respectively. The lactate dehydrogenase (LDH) release kit was purchased from Clontech Laboratories, Inc. (Mountain View, CA, USA), and the ApopTag^®^ Red *In Situ* Apoptosis Detection Kit was purchased from MilliporeSigma. The GFP-LC3 fusion plasmid was kindly provided by Dr. Noboru Mizushima. The full-length rat LC3 cDNA was inserted into pEGFP-C1, a GFP fusion protein expression vector (Kabeya et al., 2000) (Clontech Laboratories Inc, Mountain View, CA, USA), and the plasmid was verified by sequencing analysis at a core laboratory at UT Southwestern Medical Center.

### PCR Analyses for Genotyping

Genotypes were screened by isolating genomic DNA from tail biopsies and testing for transgenic sequences by PCR. For detection of Atg5, the primer sequences used were 5′-AGC GGC CTT TCA TCC AGA AG-3′ (sense) and 5′-AGA GGG GTT TCC AGC ATT GG-3′ (antisense), and for Cre, the primer sequences used were 5′-ATG CTT CTG TCC GTT TGC CG-3′ (sense) and 5′-CCT GTT TTG CAC GTT CAC CG-3′ (antisense). The thermal cycling profile consisted of denaturation at 94°C for 2 min followed by 35 cycles of denaturation at 94°C for 30 s, annealing at 60°C (Atg5) or 58°C (Cre) for 30 s and extension at 72°C for 1 min, with a final extension at 72°C for 7 min and hold at 4°C.

### Biochemical Assessment and Kidney Histology Analysis

Plasma creatinine was analyzed as described previously in the literature (Zinellu et al., 2005; Rutkowski et al., 2013; Shi et al., 2018). Kidney tissue was fixed in 4% formaldehyde for 24 h at 4°C and embedded in paraffin blocks. Sections were cut at 4 μm, stained with hematoxylin and eosin, and examined using an imaging system (Carl Zeiss Micro-Imaging, NY) by a nephropathologist blinded to the experimental protocol.

### Cell Culture

Rat renal proximal tubular epithelial (NRK) cells and opossum kidney (OK) cells were maintained in high-glucose DMEM (Gibco, USA) supplemented with 10% fetal bovine serum (MilliporeSigma) and 1% penicillin–streptomycin solution (HyClone, USA) in 5% CO_2_ at 37°C. RA was dissolved in DMSO, and cisplatin was dissolved in normal saline; both solutions were filtered with a 0.22-μm membrane filter. The concentration of RA was chosen according to the literature (Brigger et al., 2015). Cell culture supernatants were collected for measurement of LDH. Cell lysates were made and subjected to immunoblotting.

OK cells were seeded on glass coverslips and transiently transfected with GFP-LC3 plasmid using Lipofectamine 2000 (Invitrogen, USA) according to the manufacturer’s instructions. We chose OK cells as the transfection rate is higher than NRK cells. Following transfection, cells were treated with rapamycin (0.5 μM) or cisplatin (5 μM) with or without RA (1 μM) for 24 h and fixed with 4% paraformaldehyde, and fluorescence microscopy analysis was performed. The number of autophagy puncta was quantified by confocal microscopy (Nikon, Japan). Experiments were performed in triplicate for all *in vitro* studies.

### LDH Assay

NRK cells were treated with cisplatin (0–100 μM) for 12 h and 24 h in 6-well plates. Then, cells were treated with cisplatin (0–20 μM) with or without RA (1 μM) for 24 h in 12-well plates. Cells were also treated with 5 μM cisplatin plus 0.5 μM rapamycin or 10 mM LiCl or 200 nM Baf in 12-well plates. Cell culture media samples were collected and centrifuged, and the cell supernatants were used for LDH assay according to the kit instructions. The assay was performed in a 96-well plate, and the amount of LDH enzyme activity was measured by reading the absorbance of the samples at 490/492 nm with a microplate reader.

### Immunoblotting

Cultured NRK cells and frozen kidney tissue that had been stored in a −80°C freezer were lysed with a RIPA lysate mixture supplemented with EDTA-free Protease Inhibitor Cocktail (MilliporeSigma) and phosphatase inhibitor Cocktail (MilliporeSigma). Kidney tissue was soaked in the RIPA mixture and lysed with a tissue homogenizer. The Bradford assay reagent and cuvettes, which were used to measure the concentration of the protein samples, were bought from Bio-Rad, and BSA (MilliporeSigma) was used as a protein standard. All the protein samples were adjusted to the same concentration with RIPA mixture and protein loading buffer after measuring the concentration. Western blot analysis was performed as described previously (Wang et al., 2005). After blocking nonspecific binding sites with 5% BSA for 1 h at room temperature, membranes were incubated with primary antibodies against p62 (1:1,000), LC3 (1:500), Atg5(1:500), cleaved-caspase-3 (1:1,000), NGAL (1:1,000), and *β*-actin (1:1,000) overnight at 4°C followed by a corresponding secondary antibody (1:5,000) for 1 h at room temperature according to the source of the primary antibody. Signals were detected with a chemiluminescence system (GE Healthcare, Waukesha, WI, USA), and the intensities of the target bands were quantified by using ImageJ software (NIH, USA).

### Immunofluorescence

Mouse kidney paraffin slides were dewaxed and rehydrated with xylene and a 100–50% graded ethanol series. After antigens were retrieved and blocked, the slides were incubated with primary goat anti-NGAL antibody (1:500) overnight at 4°C followed by 2 h of incubation with donkey anti-goat IgG, Alexa Fluor 568 (1:200) and LEL (1:200) at room temperature. LEL was used to stain renal tubulars. After counterstaining with DAPI (10 µg/ml) for 10 min, the slides were mounted with glass coverslips with fluorescence gel (Electron Microscopy Sciences, England), and ultimately, they were analyzed under a confocal microscope (Nikon, Japan). Positive renal tubules were counted from 100 renal tubules and expressed as the percentage of positive renal tubules using NIS-Elements Imaging Software.

### Terminal Deoxynucleotidyl Transferase-Mediated dUTP Nick End Labeling

NRK cells were seeded on glass coverslips, and when confluent, they were pretreated with RA (1 µM) 30 min before adding cisplatin (10 µM) for 24 h in 6-well plates. The vehicle group was treated with the same volume of DMSO as the RA group, and the cisplatin group was treated with cisplatin (10 µM) for 24 h in 6-well plates. Cells were fixed with 4% formaldehyde for 10 min, and then the ApopTag Red in Situ Apoptosis Detection Kit protocol for adherent cultured cell types was followed as previously reported (Mohan et al., 2015). Then, the cells were analyzed under a confocal microscope (Nikon, Japan) using NIS-Elements Imaging Software.

### Statistical Analyses

Data are expressed as means ± SDs. Statistical analyses were performed using one-way ANOVA followed by the least-significant difference test for equal variance or Dunnett’s T3 tests for heterogeneity of variance using SPSS 22.0 software. P values < 0.05 were considered as indicative of statistical significance. All the graphs were made by GraphPad Prism 6.0.

## Data Availability Statement

The datasets generated for this study are available on request to the corresponding author.

## Ethics Statement

The animal study was reviewed and approved by Institutional Animal Care and Use Committee at the University of Texas Southwestern Medical Center.

## Author Contributions

JW: conception and design, collection and assembly of data, data analysis and interpretation, manuscript writing. CZ: conception and design, collection of data, data analysis and interpretation. XW: data analysis and interpretation. MS: animal experiment guidance and data collection. KM: collection of data of animal study. JM: collection of data of animal study. CC: conception and design, financial support, data analysis and interpretation, manuscript writing, final approval of the manuscript.

## Conflict of Interest

The authors declare that the research was conducted in the absence of any commercial or financial relationships that could be construed as a potential conflict of interest.

## Supplementary Material

Figure S1 Characterization of the Atg5^flox/flox^:Cagg-Cre mice model. **(A)** Breeding protocol for generating Atg5^flox/flox^:Cagg-Cre mice. **(B)** Representative images of PCR-based genotyping. **(C)** Whole-tissue lysates of kidney were collected from Atg5^flox/flox^, Cagg-Cre and Atg5^flox/flox^:Cagg-Cre mice for immunoblot analysis of Atg5, p62, LC3-II, LC3-I and β-actin. **(D)** Densitometry of Atg5, p62, and LC3-II/I signals. Data in D are expressed as the means ± SDs, *P < 0.05, **P < 0.01.
