# Supplementary figures and images for: Retinoic Acid Alleviates Cisplatin-Induced Acute Kidney Injury Through Activation of Autophagy
Source: Front Pharmacol. 2020 Jul 3;11:987. doi: 10.3389/fphar.2020.00987 (PMC7348052; doi:10.3389/fphar.2020.00987)

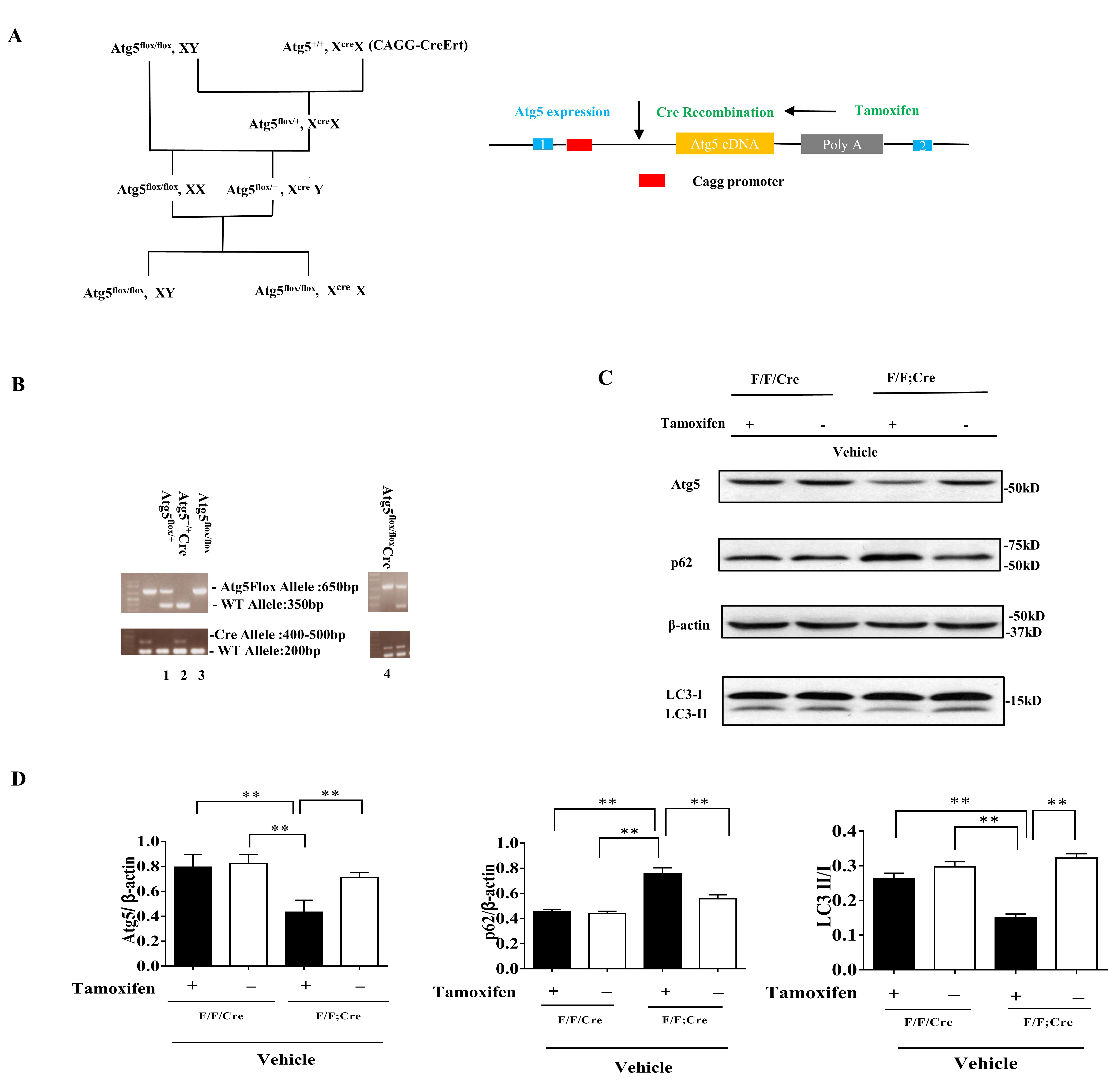

Supplement: Figure S1 — Characterization of the Atg5flox/flox:Cagg-Cre mice model. (A) Breeding protocol for generating Atg5flox/flox:Cagg-Cre mice. (B) Representative images of PCR-based genotyping. (C) Whole-tissue lysates of kidney were collected from Atg5flox/flox, Cagg-Cre and Atg5flox/flox:Cagg-Cre mice for immunoblot analysis of Atg5, p62, LC3-II, LC3-I and β-actin. (D) Densitometry of Atg5, p62, and LC3-II/I signals. Data in D are expressed as the means ± SDs, *P < 0.05, **P < 0.01. [file Image_1.tif]
