# Supplementary material for: Retinoic Acid Alleviates Cisplatin-Induced Acute Kidney Injury Through Activation of Autophagy
Source: Front Pharmacol. 2020 Jul 3;11:987. doi: 10.3389/fphar.2020.00987 (PMC7348052; doi:10.3389/fphar.2020.00987)
Supplement: Supplementary file 2 [file DataSheet_1.docx]

Cagg-Cre-ER^TM^ mice carry the Cre-recombinase protein, which comprises the Cre-recombinase protein fused to a mutant form of the mouse estrogen receptor under the Cagg promoter. The fusion protein is restricted to the cytoplasm, and Cre-ER^TM^ will have the access to the nucleus only after binding to tamoxifen. Atg5^flox/flox^:Cagg-Cre mice were given 50 mg/kg tamoxifen via intraperitoneal injection for 5 consecutive days to activate Cagg-Cre recombinase in order to knock down Atg5. (**Figure S1A right panel**). Atg5^flox/flox^ mice were bred to transgenic mice expressing Cagg-Cre recombinase to obtain Atg5^flox/+^Cre mice. Atg5^flox/+^Cre mice were then bred to Atg5^flox/flox^ mice to generate Atg5^flox/flox^Cre mice (**Figure S1A left panel**). Two rounds of PCR were conducted to verify the genotypes. Atg5^flox/+^ was shown by the amplification of a 650 bp fragment of the Atg5 flox allele, the amplification of a 350 bp fragment of the WT allele plus the absence of a 400-500 bp fragment of the Cre allele, and the amplification of a 200 bp fragment of the WT allele (**Figure S1B, lane 1**). Atg5^+/+^Cre was shown by the absence of a 650 bp fragment of the Atg5 flox allele and the amplification of a 350 bp fragment of the WT allele, a 400-500 bp fragment of the Cre allele, and a 200 bp fragment of the WT allele (**Figure S1B, lane 2**). Atg5^flox/flox^ was shown by the amplification of a 650 bp fragment of the Atg5 flox allele, the absence of both the 350 bp fragment of the WT allele and the 400-500 bp fragment of the Cre allele, and the amplification of a 200 bp fragment of WT allele (**Figure S1B, lane 3**). Atg5^+/+^Cre and Atg5^flox/flox^ mice were used as control mice in the experiment. Atg5^flox/flox^Cre mice were shown by the amplification of a 650 bp fragment of the Atg5 flox allele, a 400-500 bp fragment of the Cre allele and a 200 bp fragment of WT allele (**Figure S1B, lane 4**). After tamoxifen treatment, basal autophagy was lower in the Atg5^flox/flox^Cre group with tamoxifen treatment than in the Atg5^flox/flox^Cre group without tamoxifen treatment and the control groups with or without tamoxifen treatment as shown by an increased level of p62, a decreased level of Atg5 and a decreased ratio of LC3-II/LC3-I in the kidney tissue detected by immunoblotting (**Figure S1C, D, P<0.01**).

Figure legend

**FIGURE S1** Characterization of the Atg5^flox/flox^:Cagg-Cre mice model. **(A)** Breeding protocol for generating Atg5^flox/flox^:Cagg-Cre mice. **(B)** Representative images of PCR-based genotyping. **(C)** Whole-tissue lysates of kidney were collected from Atg5^flox/flox^, Cagg-Cre and Atg5^flox/flox^:Cagg-Cre mice for immunoblot analysis of Atg5, p62, LC3-II, LC3-I and β-actin. **(D)** Densitometry of Atg5, p62, and LC3-II/I signals. Data in D are expressed as the means ± SDs, *P < 0.05, **P < 0.01.
